# First person – Ronan Smith and Nicole Reyne

**DOI:** 10.1242/dmm.052735

**Published:** 2025-12-29

## Abstract

First Person is a series of interviews with the first authors of a selection of papers published in Disease Models & Mechanisms, helping researchers promote themselves alongside their papers. Ronan Smith and Nicole Reyne are co-first authors on ‘ Mapping lung cancer ventilation dynamics using functional imaging and lung mechanics’, published in DMM. Ronan is a postdoc in the lab of Professor Martin Donnelley at University of Adelaide, Adelaide, Australia, investigating novel methods and applications of X-ray imaging. Nicole is a postdoctoral researcher in the same lab, investigating preclinical models of lung diseases.


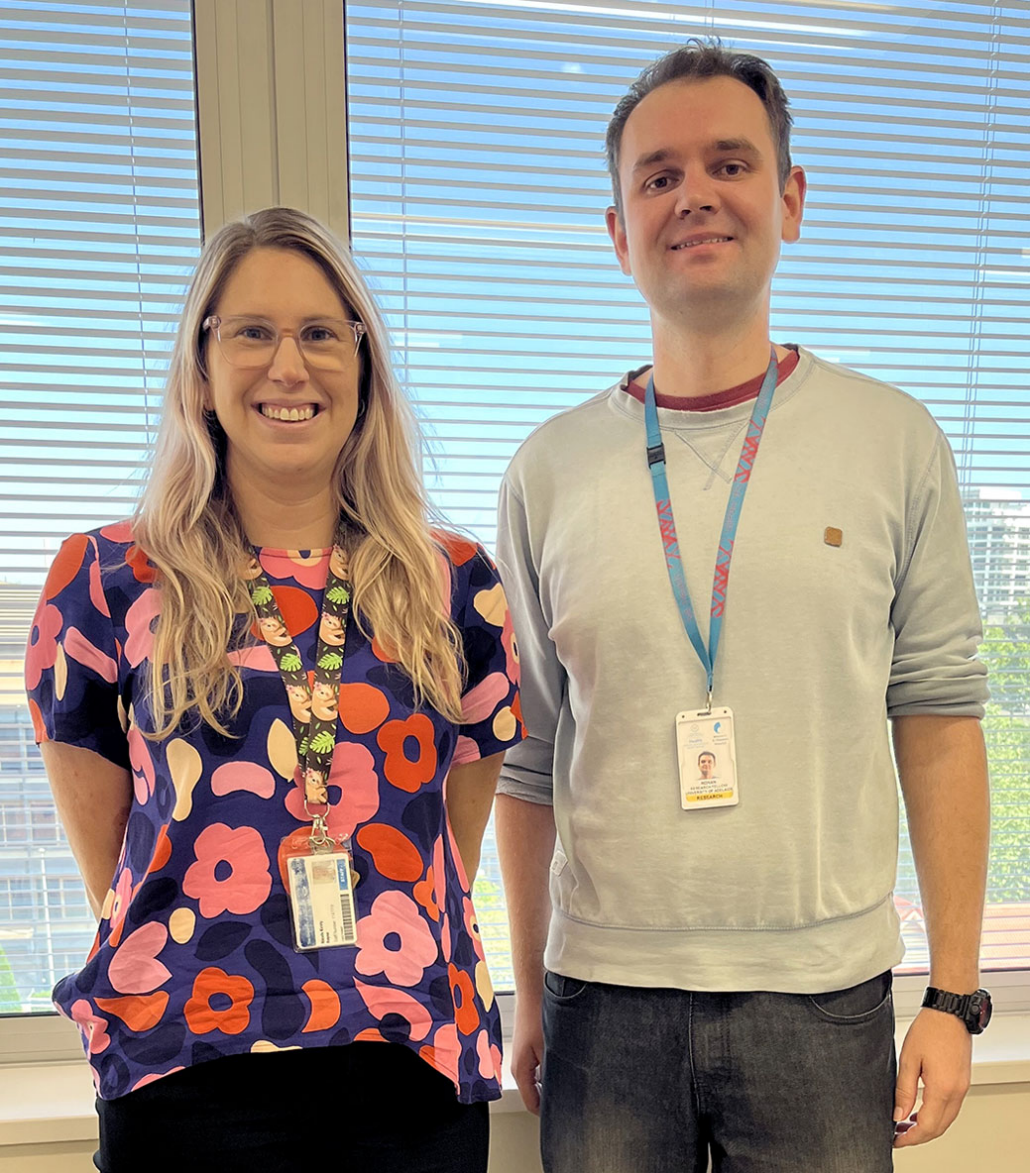


**Ronan Smith (right) and Nicole Reyne (left)**

**Who or what inspired you to become a scientist?**

**R.S.:** I have always enjoyed solving problems and always looked for interesting opportunities, so deciding to do a PhD was an easy choice. From there, I saw a job advert to come and apply my physics skills in biomedical research, which has been a great learning experience. Along the way I have worked with a number of great scientists who have motivated and encouraged me.

**N.R.:** I've always loved working with animals, which led me to attend an agricultural high school and complete a degree in animal science. Working in an animal facility after that introduced me to the cystic fibrosis rat colony, and that's how I joined the research group. Since then I have been running the preclinical studies and managing our rat and mouse colonies, which has been very rewarding.

**What is the main question or challenge in disease biology you are addressing in this paper? How did you go about investigating your question or challenge?**

**N.R.:** This paper addresses how lung function and ventilation are altered in the presence of lung disease (cancer) in mice, specifically looking at regional changes in the lung that are not easily captured by global lung function tests alone. We did this by combining a relatively new functional lung imaging technology called X-ray velocimetry (XV) with traditional lung function testing (flexiVent) in a mouse model of lung cancer. XV allowed us to visualise and quantify regional ventilation changes across the lung, while flexiVent provided global measures of lung mechanics such as compliance, resistance and inspiratory capacity.

**How would you explain the main findings of your paper to non-scientific family and friends?**

**R.S.:** In our study, we looked at how lung function changes during cancer in mice. Normally in the clinic, overall lung function is measured using spirometry, where the patient blows into a device that measures airflow. However, spirometry doesn't say where in the lung any changes are coming from. So in our study we combined a mouse version of spirometry with a novel imaging technology called XV that lets us see how air moves through different parts of the lungs and pinpoint the location of any abnormalities. We showed that this could be used to track how airflow changes in the lung as cancer develops. Although our study used mice, we think that XV could help people with lung cancer.

[Our study] results provide a more detailed understanding of how lung disease affects regional lung function

**What are the potential implications of these results for disease biology and the possible impact on patients?**

**R.S.:** The XV imaging method we used in the paper is really exciting, with future studies hopefully able to more accurately measure changes to lung function when testing treatments. Our research group is also running the world's first paediatric clinical trial of XV imaging, looking at identifying ventilation defects in children with cystic fibrosis.

**N.R.:** These results provide a more detailed understanding of how lung disease affects regional lung function, which is critical for understanding disease progression. For patients, this could eventually translate to better monitoring of lung health, earlier detection of functional impairments and more targeted therapies, as we can identify which areas of the lung are most affected and track changes over time. Further, we can apply this to other lung diseases, like asthma, cystic fibrosis and chronic obstructive pulmonary disease.

**Figure:**
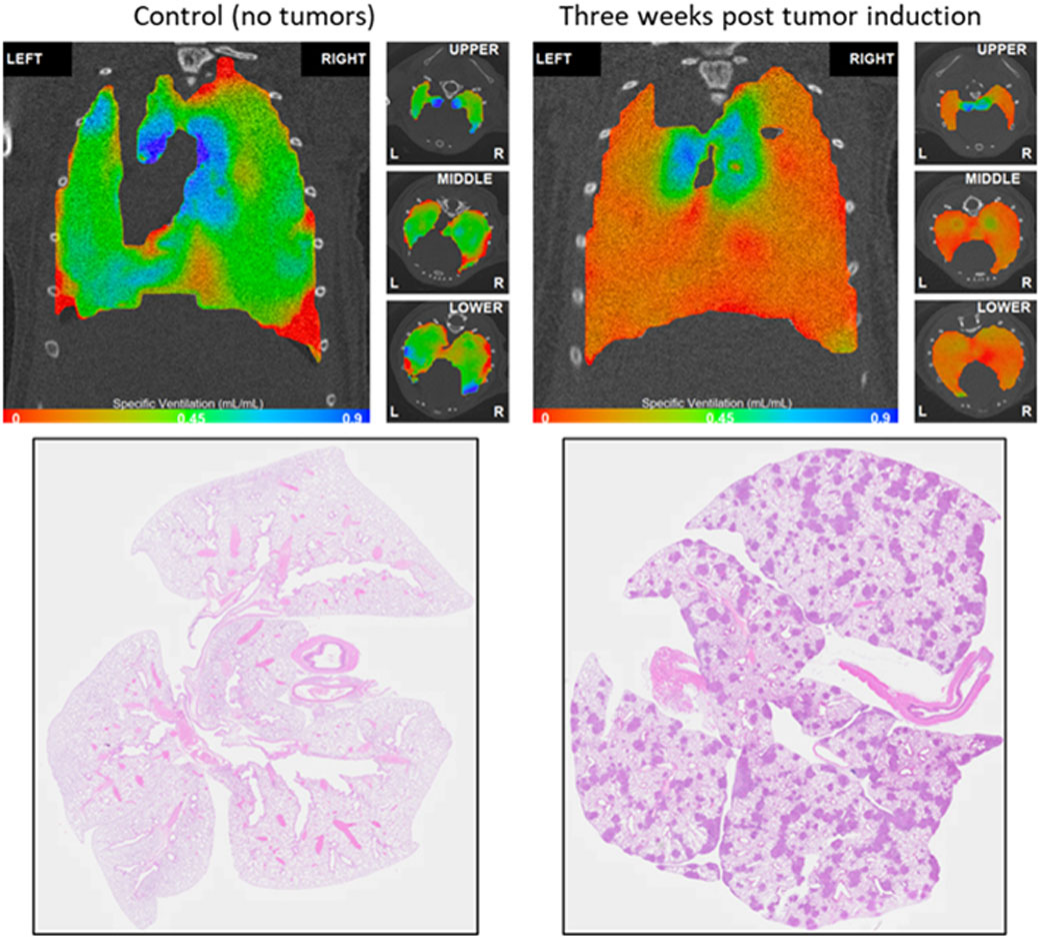
**Comparison of a healthy control lung and a lung 3 weeks post tumour induction.** This comparison is very striking as the red colouring in the 3 weeks post tumour induction lung indicates low ventilation, meaning that those lungs are under ventilated and not working well. The histology image shows the location of the tumours causing the ventilation defects.

**Why did you choose DMM for your paper?**

**R.S./N.R.:** A disease model was the main focus of the paper, so we thought it fit the journal nicely. Additionally, Adelaide University has a Read & Publish Agreement with DMM, which meant we could save our funding for more research by publishing here as the University has already covered the fee. Transferring our *bioRxiv* preprint into the journal was also a really easy process.

**Given your current role, what challenges do you face and what changes could improve the professional lives of other scientists in this role?**

**R.S./N.R.:** We're both early-career researchers, so neither of us have permanent positions, with our jobs reliant on grant funding to extend our employment contracts. More funding opportunities and more opportunities to gain permanent contracts would be a huge benefit, but this would need government policy changes and a change in attitude towards research.

A challenge that we are able to work towards solving is the recognition of the value of interdisciplinary work. By combining our skillsets we were able to do some good work here, but we still struggle sometimes to recruit students from across faculties who could bring in new skills.

**What's next for you?**

**R.S.:** I am looking at how computer vision and machine learning can help us dig deeper into the XV data we showed here, giving a better understanding of what is happening in the lung during different diseases and helping diagnostic accuracy.

**N.R.:** What's next for me is looking at how lung ventilation changes over time. So far, we've mainly captured a single snapshot using XV, but I'm really interested in tracking lung ventilation across multiple timepoints to see how different diseases affect lung health as they progress. Eventually, I'd like to use this approach to study how treatments change lung ventilation too.

**Tell us something interesting about yourself that wouldn't be on your CV**

**N.R.:** Outside of research I am a keen long distance runner, and the longest run I have completed was 176 km through the Blue Mountains near Sydney, Australia. It's a very hilly area too!

**R.S.:** My main hobby of whitewater kayaking was interrupted when I moved from the rainy UK to the driest city in Australia (a country already known for its lack of rain), but I still enjoy kayaking and paddleboarding on the beaches here.
